# The Kessler 6 psychological distress scale (K6) in swedish: a psychometric study of reliability, factor structure and measurement invariance in a large population-based sample

**DOI:** 10.1186/s40359-026-05857-9

**Published:** 2026-10-23

**Authors:** Karolina Ehnsiö, Sanna Tiikkaja, Johnny Pellas

**Affiliations:** 1 Division of Primary Care, Region Sörmland, Eskilstuna, Sweden; 2 Social Medicine/CHAP, Department of Public Health and Caring Sciences, Uppsala University, Uppsala, Sweden; 3 Research and Development Unit, FoU i Sörmland, Region Sörmland, Eskilstuna, Sweden; 4 Clinical Geriatrics, Department of Public Health and Caring Sciences, Uppsala University, Uppsala, Sweden

**Keywords:** Poor mental health, Validation, Screening tool, Psychiatric disorders, Psychological testing

## Abstract

**Background:**

Psychological distress is a major public health concern, and brief, reliable screening instruments are essential for early detection and monitoring. The six-item Kessler Psychological Distress Scale (K6) is widely used internationally; however, evidence regarding its psychometric properties in Sweden remains limited. This study examined the reliability, factor structure, and measurement invariance of the Swedish version of the K6 in a large population-based sample.

**Methods:**

Data were obtained from the Life and Health Survey (2022), conducted in five regions in Mid-Sweden. The study included 31,759 adults aged 18–102 years. Internal consistency was assessed using McDonald’s omega total. Structural validity was evaluated using confirmatory factor analysis, comparing a one-factor model, a two-factor model representing depression and anxiety, and a bifactor model comprising a general psychological distress factor and two specific factors representing depression and anxiety. Measurement invariance of one-factor model was examined across age groups and by gender using multigroup CFA, testing configural and metric invariance.

**Results:**

Internal consistency was high across all demographic groups (McDonald’s omega total 0.883–0.899). The one-factor model demonstrated best fit, with acceptable values according to CFI, TLI, and SRMR, but unacceptable according to RMSEA (CFI = 0.996, TLI=0.993, RMSEA=0.069, SRMR=0.020), with standardized loadings ranging from 0.70 to 0.91. The two-factor model showed acceptable fit according to CFI, TLI and SRMR, but unacceptable fit according to RMSEA (CFI=0.989, TLI=0.982, RMSEA=0.109, SRMR=0.040). Standardized factor loadings were high for both factors (Factor 1: 0.78; Factor 2: 0.78–0.91). The correlation between the two latent factors was extremely strong (*r*=.99). The bifactor model did not converge and therefore could not be evaluated or interpreted further. Configural and metric invariance were supported across the age groups and between men and women.

**Conclusions:**

The Swedish version of the K6 demonstrates strong reliability and supports a unidimensional structure. Measurement invariance testing supported comparable measurement of psychological distress across age and gender. The scale is suitable for monitoring psychological distress in public health surveys and may also be useful as a brief screening tool in clinical practice. Further research should establish clinically relevant threshold values in a Swedish context.

**Trial registration:**

Not applicable.

## Introduction

Mental health problems encompass a wide range of conditions, varying in severity and duration, from mild and transient issues to long-term conditions with significant functional impairment [1]. The term can thus include everything from mild states of sadness or anxiety to psychiatric disorders such as depression, anxiety disorders, or schizophrenia. Globally, mental health problems are currently among the leading causes of disease burden and healthcare costs [2, 3]. Screening for mental health problems in the population can therefore be considered an important public health initiative that contributes to early detection, preventive efforts, and efficient resource allocation. Reliable, culturally adaptable, and time-efficient assessment tools are crucial for effective diagnosis and intervention both in clinical practice and at the population level [4].

Due to their efficiency and psychometric strength, the Kessler psychological distress scales with 10 and 6 items (K10 and K6) are frequently used today for screening mental health problems [5, 6]. The K6 has proven effective in identifying both moderate psychological distress and more severe mental health issues, depending on the threshold used [7, 8]. In Sweden, the K6 is currently included in the National Public Health Survey Health on Equal Terms (HET) as well as in the Life and Health Survey (LH) used in Mid- Sweden [9, 10]. Both surveys are conducted regularly and aim to provide an overview of the public health status of Sweden’s population and to monitor changes in health and its determinants over time. A meta-analysis of the K6 emphasizes the importance of careful adaptation and validation processes to ensure reliability across different linguistic and cultural contexts [4]. To our knowledge, only one study has previously assessed psychometric properties on the K6 in a Swedish context [11]. Lundin et al. (2024) investigated the comparability of the K6 and the 12-item General Health Questionnaire (GHQ-12) and found that the two instruments appeared to measure the same underlying construct of psychological distress. Using bifactor analysis, Rasch analysis, and equipercentile equating, they provided evidence of construct validity and demonstrated that scores on the two scales could be translated between each other.

Numerous studies internationally have investigated the reliability of K6. Cronbach’s α usually ranges between 0.80 and 0.89, indicating good internal consistency and reliability across different populations [4].

The K6 was developed to assess non-specific psychological distress, a construct reflecting emotional suffering and symptoms that are common across depressive and anxiety disorders [5, 6]. From this perspective, the K6 is expected to function as a unidimensional measure of general psychological distress. However, the scale includes items reflecting experiences typically associated with anxiety (i.e., nervousness and restlessness) as well as depression (i.e., hopelessness, depressed mood, worthlessness, and feeling that everything was an effort). Previous research on the factor structure of the K6 has shown mixed findings [7, 12–17]. Several studies using confirmatory factor analysis (CFA) have supported a unidimensional model, suggesting that the scale primarily measures general psychological distress [12, 16, 18]. For example, Umucu et al. demonstrated that both exploratory and confirmatory factor analyses consistently supported a unidimensional factor structure of the K6, with all items loading on a single underlying psychological distress construct [12]. Sunderland et al. reported acceptable fit for a single-factor model, despite minor item bias regarding age differences [13]. Other studies have, however, identified more complex structures. Carter et al. demonstrated that a bifactor model, comprising a general distress factor alongside specific factors for anxiety and depression, provided the best fit in a sample of adults with severe and persistent mental illness [14]. Similarly, Bessaha et al. found that among emerging adults, a two-factor model separating anxiety and depressive symptoms provided a better fit than a one-factor model, although all models demonstrated acceptable fit [15]. Mewton et al. also found support for a one-factor model but noted that differences in item thresholds between genders may reflect reporting bias rather than true differences in underlying psychological distress [16]. These discrepancies underscore the importance of analytic approach and choice of fit criteria in drawing conclusions about factorial validity. Although the K6 generally performs well as a unidimensional instrument, demographic and cultural variables, as well as the implications of model selection and fit thresholds, should be considered when evaluating its factor structure.

Previous studies have demonstrated that the K6 scale exhibits strong construct validity across genders, despite certain symptoms being expressed differently by females and males over time [19]. The higher average level of psychological distress observed among females appears to reflect a genuine difference rather than a gender-related measurement bias. Furthermore, a previous study has also found measurement noninvariance by age, with acceptable model fit for younger but not older adults [14].

Lundin et al. show in their study that the Swedish versions of K6 and the General Health Questionnaire 12 (GHQ-12) seem to measure the same underlying construct based on bifactor analysis [11]. However, the study did not primarily evaluate the psychometric properties of the K6 itself, such as its internal consistency reliability or factorial structure within a Swedish population. Consequently, further validation of the Swedish K6 remains warranted. The present study addresses this gap by examining the scale’s reliability and structural validity in a large population-based Swedish sample, thereby providing complementary evidence regarding the psychometric performance of the instrument.

## Methods

### Participants

This study includes 31 759 individuals who answered the questions of K6 in the LH. Five regions of Mid-Sweden (Uppsala, Värmland, Västmanland, Sörmland and Örebro) collaborated on the data collection together with Statistics Sweden (SCB). The Swedish Total Population Register was used for the sampling frame, and a stratified random sample by age-group, gender, and municipality/county was drawn by SCB. In total, 78 117 people, aged 18 years or older, in the five counties received the postal survey, which could be answered by paper/mail or on-line. The sample represents the population of Mid-Sweden, consisting of 1,2 million persons living in these five regions. The data collection was carried out in the spring 2022, and it was discontinued after three reminders were sent via SMS, to those responders who had a registered mobile phone number. Otherwise, the data collection was discontinued after two postal reminders, however males in the youngest group (18–29 years) received an additional postal reminder. The overall response rate for LH 2022 was 45%, and the variations between the regions were small (43–47%). The survey includes questions on health, living conditions as well as health-related behaviours. Additional information of the respondents’ gender, age, country of birth, and educational level were retrieved from the population registers. The data from questionnaires and the registries were merged and pseudonymized by SCB, before delivery for further processing in the regions of Mid-Sweden. The survey, initial descriptive analyses, and “dropout” analysis presented in Swedish public health reports [10, 20]. Several demographic variables were included in this study to describe the population and to assess possible variations in K6. Gender included females/males and age was categorized into 10-year groups (18–29, 30–39, 40–49, 50–59, 60–69, 70–79, 80–89, 90+). Highest educational level was categorized as “pre-secondary,” “upper-secondary” and “post-secondary.” Birth country included “Sweden,” “other Nordic countries” and “other country.”

### Measures

The K6 consists of six items, measuring the respondents’ mental health symptoms during the last 30 days: feeling (1) nervous, (2) hopeless, (3) restless or fidgety, (4) so depressed that nothing could cheer you up, (5) that everything was an effort, and (6) worthless. For each of these questions, a score of 0, 1, 2, 3, or 4 is assigned with the following response options: “none of the time,” “a little of the time,” “some of the time,” “most of the time,” or “all of the time,” respectively. The responses to these six items are summed to yield a K6 score of 0–24, and higher scores indicate high levels of psychological distress [5]. Those scoring < 5, ≥ 5 to < 13, and ≥ 13 are categorized as having no/low, moderate, and serious psychological distress, respectively [8]. The scales are free to use without permission (available here: https://rckessler.scholars.harvard.edu/k10-and-k6-scales). This study used the official Swedish version of the K6, translated by the Centre for Epidemiology and Community Medicine in Stockholm, Sweden [11].

### Statistical analyses

The demographic characteristics of the study population were described using age groups and are presented as proportions (%), means, and standard deviations (SD). Total score distribution was examined by gender. Mean K6 scores and standard deviations were examined by gender and age group. Internal consistency reliability of the K6 was assessed using McDonald’s ω total and interpreted according to commonly used guidelines, with values between 0.65 and 0.80 indicating acceptable reliability and values above 0.80 indicating strong reliability [21]. McDonald’s ω was chosen because it does not require the assumption of equal factor loadings across items and is therefore considered more appropriate when items may contribute unequally to a common latent construct. Structural validity was investigated through CFA. Three competing models were tested: a one-factor model as suggested by previous research [12, 16, 18], a two-factor model suggested by others [15, 17] with a depression factor (items 2, 4, 5 and 6) and an anxiety factor (items 1 and 3), and a bifactor model comprising a general psychological distress factor and two orthogonal specific factors representing depression (items 2, 4, 5, and 6) and anxiety (items 1 and 3) as suggested by previous research [14]. In the two-factor model, the anxiety factor was specified by two indicators (items 1 and 3). To ensure model identification in the two-factor model, the factor loadings of the two items in the anxiety factor were constrained to equality. The factor loadings in the depression factor were freely estimated. The models were estimated using the weighted least squares mean- and variance-adjusted estimator (WLSMV), which is suitable for CFA with ordinal data. Model fit was evaluated using the scaled (i.e., robust) versions of the chi-square statistic (χ²) with degrees of freedom (df), comparative fit index (CFI), Tucker–Lewis index (TLI), root mean square error of approximation (RMSEA) and was also evaluated with the standardized root mean square residual (SRMR). The following criteria were used to guide interpretation of acceptable model fit: RMSEA < 0.06; SRMR < 0.08; CFI and TLI > 0.95 [22]. Because conventional fit-index cut-offs were developed primarily for continuous-data models estimated using maximum likelihood and may not be directly applicable to ordinal CFA estimated using WLSMV, the fit indices were interpreted as general guidelines rather than strict decision rules. Measurement invariance of the K6 model with best fit was subsequently examined across age groups and by gender using multigroup CFA. Configural and metric invariance were tested sequentially. Configural invariance assessed whether the same factor structure held across groups, while metric invariance was tested by constraining factor loadings to equality across groups. Changes in CFI (ΔCFI) and RMSEA (ΔRMSEA) between models were used to evaluate measurement invariance, with ΔCFI ≤ 0.020 and ΔRMSEA ≤ 0.030 indicating metric invariance [23]. CFAs and analyses of measurement invariance were conducted using the SEM-module in Jamovi 2.7.33 (available from https://www.jamovi.org). All other analyses were made using SPSS version 29.

## Results

### Descriptives

Descriptive data is displayed in Table 1. The proportions of females were larger, compared to males (54.2% vs. 45.8%). Females also held larger proportions in the age-groups below 70–79 years, whereafter the gender distribution was similar. The mean age of the study population was 58.6 years (SD 19.9), and the largest proportion belonged to the age-group 70–79 years. A majority (44.5%) had upper secondary education, thus pre-secondary education was more common among the older age-groups, while the younger age-groups often held post-secondary education. Almost nine of ten were born in Sweden.

**Table 1:** Descriptive statistics for the total sample by age groups, *n* = 31 759

	*N*	Total sample	18–29 *n* = 3,871	30–39 *n* = 2765	40–49 *n* = 3,517	50–59 *n* = 4,482	60–69 *n* = 5,366	70–79 *n* = 7,458	80–89 *n* = 3,603	90+ *n* = 697
Gender in %	31,759									
Female		54.2	60.7	60.4	59.0	54.7	53.9	50.1	46.9	49.1
Male		45.8	39.3	39.6	41.0	45.3	46.1	49.9	50.9	50.2
Age in years	31, 759									
Mean		58.6	23.7	34.6	44.8	54.7	64.7	74.3	84.2	92.4
SD		19.9	3.7	2.9	2.9	2.8	2.9	2.8	2.8	2.5
Age distribution in %	31, 759	100	12.2	8.7	11.1	14.1	16.9	23.5	11.3	2.2
Highest educational level in %	29, 498									
Pre secondary		17.6	6.0	5.3	5.4	8.1	14.4	24.7	40.4	49.9
Upper-secondary		44.5	43.6	37.0	39.9	51.0	50.0	45.9	36.7	31.1
Post secondary		38.0	50.4	57.7	54.7	40.9	34.7	29.4	23.0	18.9
Birth country in %	31, 756									
Sweden		87.8	87.7	83.7	83.8	87.0	89.2	90.1	89.0	90.1
Other Nordic country		4.0	0.5	1.0	1.6	3.4	5.3	6.3	6.4	4.3
Other country		8.1	11.8	15.2	14.6	9.6	5.5	3.6	4.6	5.6

Note. SD = Standard deviation

**Fig. 1:**
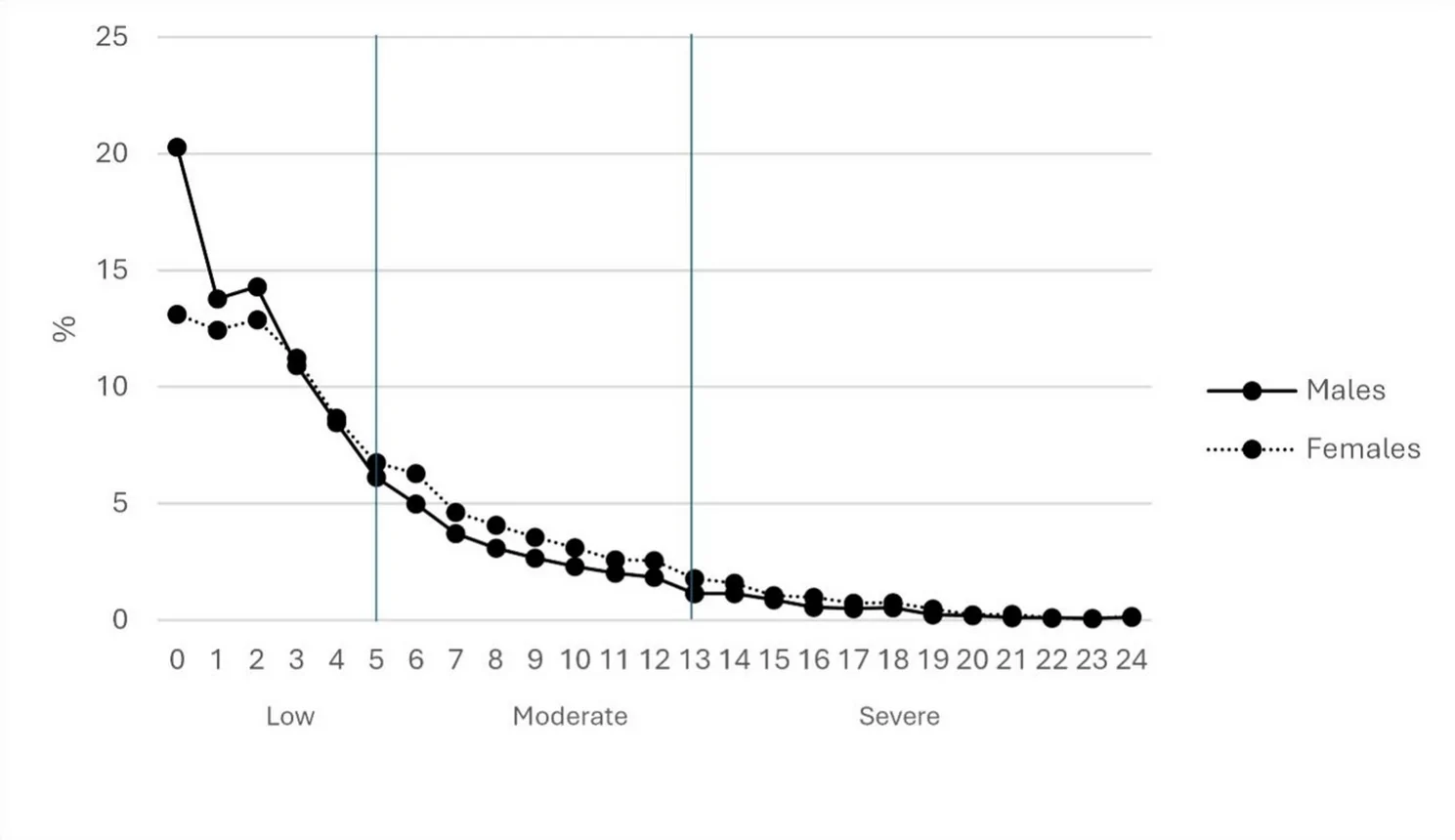
Total score distribution on the K6 by gender

Figure 1 shows the sum of Kessler 6 is not normally distributed and that males report lower scores than females. The most common score was 0 which was reported by 20.3% of males and 13.1% of females. The proportions of low psychological distress were 58.3% in females and 67.7% among males. Females reported larger proportions of moderate psychological distress (33.5%) and severe psychological distress (8.1%) compared to males (26.7% and 5.5%, respectively).

Females had higher mean scores per item and on the total score of K6 than males (4.94, SD = 4.60 vs. 3.99, SD = 4.25), as shown in Table 2. The largest mean per item was observed for feeling nervous among 17–29-year-old females (1.75, SD = 1.02), and the lowest mean was seen for feeling depressed among 60–69-year-old males (0.27, SD = 0.63). Those in the youngest age-group had the highest mean K6 scores, while the age-group 60–69 had the lowest mean scores in both genders, followed by somewhat higher mean scores in the age-group 80–89.

**Table 2:** Mean Kessler 6 scores, by item and in total

	*N*	Total sample	18–29 *n* = 3,871	30–39 *n* = 2765	40–49 *n* = 3,517	50–59 *n* = 4,482	60–69 *n* = 5,366	70–79 *n* = 7,458	80–89 *n* = 3,603	90+ *n* = 697
Nervous										
female	17, 199	1.29	1.75	1.51	1.37	1.25	1.13	1.07	1.21	1.25
SD		0.95	1.02	0.93	0.93	0.92	0.89	0.90	0.97	0.94
male	14, 560	0.97	1.32	1.23	1.14	1.01	0.83	0.79	0.91	1.03
SD		0.91	0.97	0.93	0.95	0.92	0.84	0.85	0.90	0.96
Hopeless										
female	17,199	0.58	0.91	0.66	0.57	0.52	0.43	0.47	0.64	0.78
SD		0.90	1.07	0.93	0.91	0.87	0.78	0.80	0.93	1.04
male	14,560	0.47	0.80	0.64	0.54	0.43	0.33	0.36	0.48	0.64
SD		0.85	1.06	0.98	0.91	0.82	0.70	0.73	0.84	0.99
Restless of fidgety										
female	17, 199	1.05	1.55	1.24	1.09	0.95	0.86	0.82	0.79	0.78
SD		0.78	1.07	1.00	0.98	0.95	0.87	0.88	0.94	0.94
male	14, 560	0.94	1.48	1.34	1.07	0.95	0.82	0.76	0.73	0.69
SD		0.95	1.03	0.99	0.96	0.93	0.88	0.85	0.89	0.91
Depressed										
female	17, 199	0.46	0.80	0.56	0.48	0.41	0.32	0.34	0.44	0.52
SD		0.81	0.99	0.86	0.82	0.76	0.70	0.70	0.78	0.86
male	14, 560	0.38	0.66	0.52	0.45	0.38	0.27	0.28	0.36	0.48
SD		0.76	0.93	0.85	0.82	0.78	0.63	0.65	0.73	0.85
Feeling that everything was an effort										
female	17, 199	1.01	1.47	1.22	1.09	0.98	0.76	0.79	0.96	1.06
SD		1.06	1.19	1.09	1.09	1.04	0.92	0.95	1.03	1.09
male	14, 560	0.79	1.13	1.09	0.95	0.79	0.61	0.61	0.79	0.94
		0.96	1.11	1.09	1.02	0.93	0.84	0.87	0.94	1.07
Worthless										
female	17, 199	0.57	1.00	0.78	0.58	0.48	0.37	0.39	0.57	0.76
SD		0.96	1.20	1.04	0.95	0.86	0.78	0.79	0.96	1.10
male	14, 560	0.43	0.76	0.61	0.51	0.38	0.28	0.31	0.42	0.63
SD		0.85	1.09	1.00	0.90	0.81	0.69	0.72	0.83	1.04
Mean totals score K6										
Female	17, 199	4.94	7.48	5.97	5.19	4.60	3.88	3.90	4.62	5.13
SD		4.60	5.27	4.97	4.53	4.34	3.91	4.03	4.58	4.54
Male	14, 560	3.99	6.16	5.43	4.67	3.94	3.14	3.12	3.71	4.41
SD		4.25	4.89	4.62	4.41	4.18	3.65	3.70	4.12	4.78

Note. SD = Standard deviation

### Reliability

Reliability was estimated using McDonald’s ω total across different demographic groups. The analysis consistently yielded high values, indicating strong internal consistency across all groups. The lowest reliability was observed in the 60–69 age group (ω = 0.883), whereas the highest was found in the 90 + age group (ω = 0.899). Other age groups fell within this narrow range: 18–29 years (ω = 0.891), 30–39 years (ω = 0.884), 40–49 years (ω = 0.884), 50–59 years (ω = 0.892), 70–79 years (ω = 0.885), and 80–89 years (ω = 0.894). When comparing gender strata, females exhibited a slightly higher value (ω = 0.896) than males (ω = 0.888). Overall, the findings demonstrate that the instrument has high and consistent reliability regardless of age or gender.

### Factor analysis

**Fig. 2:**
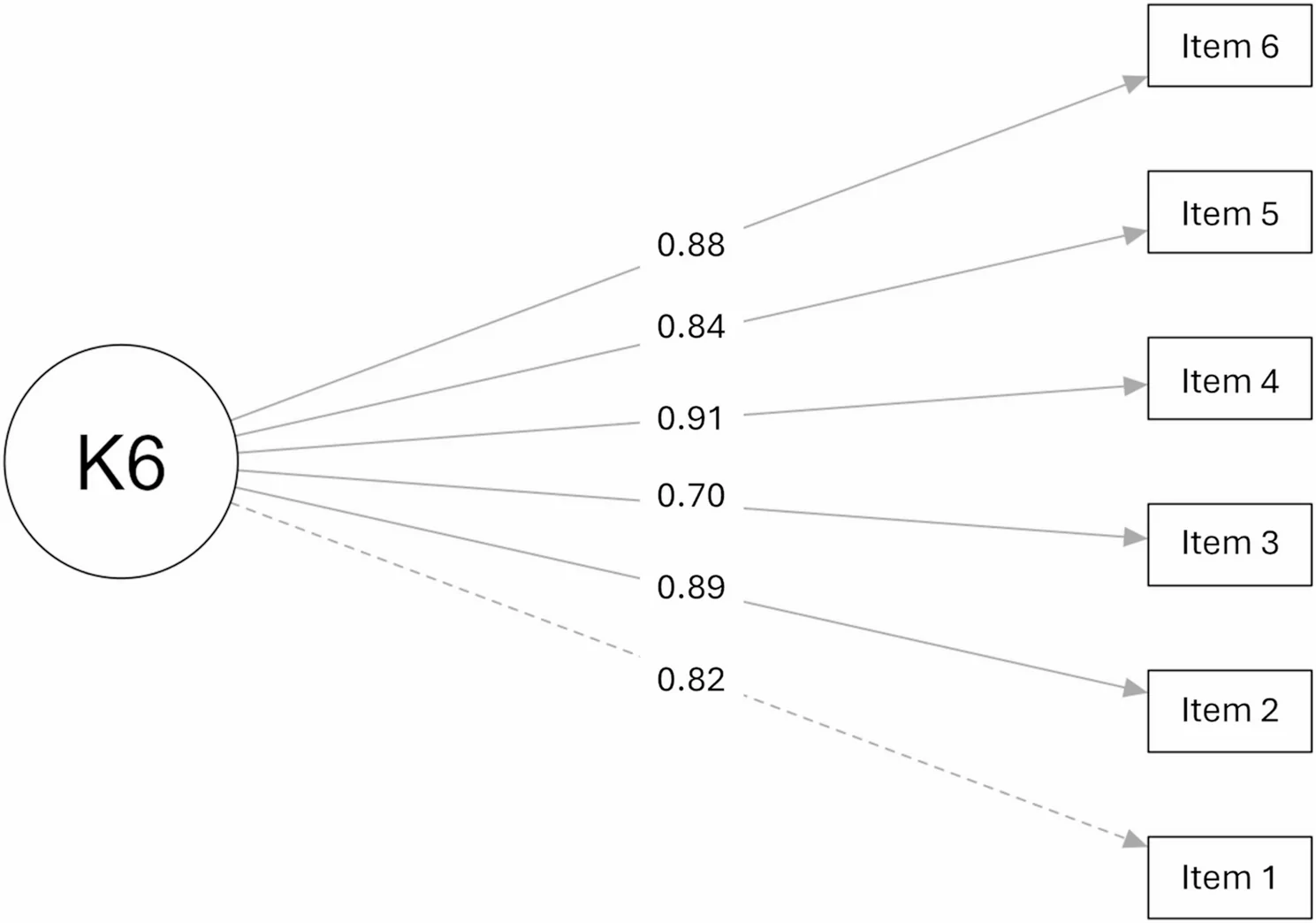
Confirmatory factor analysis of Kessler 6 with a one-factor model of psychological distress

**Fig. 3:**
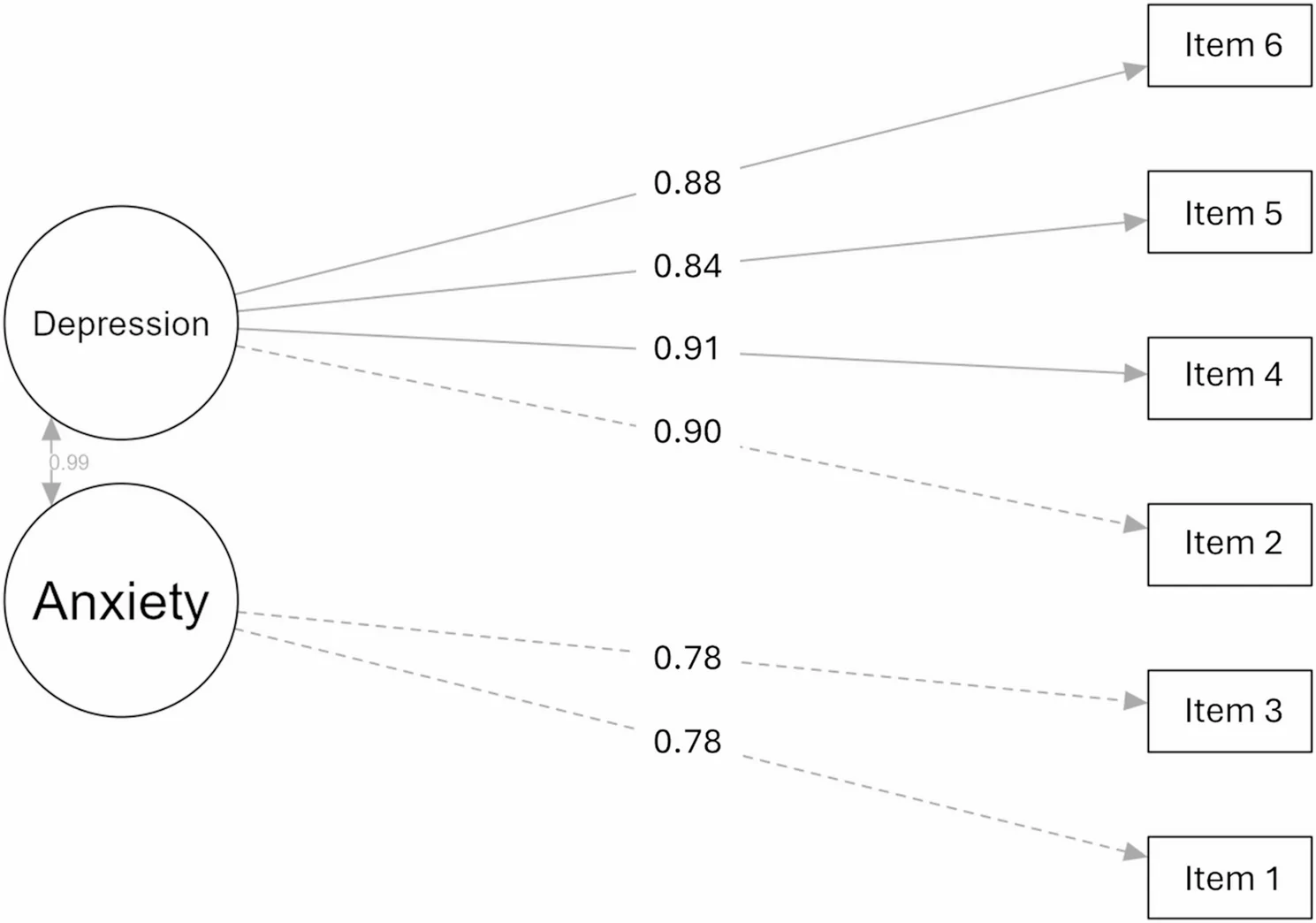
Confirmatory factor analysis of Kessler 6 with a two-factor model of anxiety and depression

The one-factor model, in which all six items loaded on a single latent factor, demonstrated acceptable fit according to CFI, TLI and SRMR, but unacceptable according to RMSEA (χ² (9) = 1383, *p* < .001, CFI = 0.996, TLI = 0.993, RMSEA = 0.069, SRMR = 0.020). All items loaded strongly and significantly on the latent factor, with standardized loadings ranging from 0.70 to 0.91 (see Fig. 2), indicating that all items were strongly related to the latent construct. The two-factor model also showed acceptable model fit according to CFI, TLI and SRMR, and unacceptable fit according to RMSEA (χ² (9) = 3392, *p* < .001, CFI = 0.989, TLI = 0.982, RMSEA = 0.109, SRMR = 0.040). All indices were worse than for the one-factor model. The standardized loadings were 0.78 for both anxiety items and ranged from 0.84 to 0.91 for the four depression items, see Fig. 3. The correlation between the two latent factors was extremely strong (*r* = .99), indicating substantial overlap between the constructs. The bifactor model comprising a general psychological distress factor and two orthogonal specific factors representing anxiety and depression was also tested. The model did not converge and therefore could not be evaluated or interpreted further.

### Measurement invariance

Across age groups, the configural model showed excellent fit for CFI, but unacceptable fit for RMSEA (CFI = 0.975, RMSEA = 0.071). Constraining factor loadings to equality resulted in a small change in fit (CFI = 0.968, RMSEA = 0.069), corresponding to ΔCFI = − 0.007 and ΔRMSEA = − 0.002, which were within the criteria for metric invariance. Measurement invariance by gender showed a similar pattern. The configural model had excellent fit for CFI, but unacceptable fit for RMSEA (CFI = 0.977, RMSEA = 0.071), and constraining factor loadings to equality did not reduce CFI and decreased RMSEA (CFI = 0.977, RMSEA = 0.049), corresponding to ΔCFI = 0.000 and ΔRMSEA = − 0.022. Thus, metric invariance was supported across gender.

## Discussion

The aim of this study was to evaluate the Swedish version of the K6 regarding reliability and factor structure in a large population-based sample. Overall, the results indicate that the K6 demonstrates strong reliability across gender and age groups and that the instrument functions primarily as a unidimensional scale for psychological distress. Furthermore, differences in levels of psychological distress between females and males were observed, as well as age-related variation, with higher mean scores among younger groups, lower scores in middle age, and a slightly higher in older age. These findings are consistent with previous psychometric studies supporting the reliability and structural validity of the K6 as a measure of psychological distress [5, 6]. Taken together, these findings suggest that the K6 is a useful and efficient measure for assessing psychological distress in Swedish public health surveys. The results also indicate potential utility in clinical contexts. However, caution is warranted because the present study did not evaluate the scale against clinical diagnoses or in clinical populations. Further research is therefore needed to establish its diagnostic performance and clinically relevant threshold values in a Swedish context.

Internal consistency was high across all age groups (ω ≈ 0.88–0.90) and across gender strata. This is consistent with previous findings [4]. The observed gender difference, with higher mean scores among females, is consistent with previous findings and suggests that the K6 captures genuine differences in reported distress rather than being primarily driven by measurement bias [16, 19]. The age-related pattern also aligns with earlier studies, although some research has indicated age-related differential item functioning (DIF) [13].

The CFAs indicated that the one-factor model provided the best fit, whereas the correlated two-factor model showed poorer fit. Although the two-factor model was statistically distinguishable from the one-factor model, its poorer fit and the very high correlation between the anxiety and depression factors provide no support for a multidimensional structure. A bifactor model with a general psychological distress factor and specific anxiety and depression factors was also examined. Unlike Carter et al. [14] who successfully estimated a bifactor model of the K6, the model did not converge in the present study and could therefore not be evaluated further. One possible explanation is the two-item anxiety factor, which provides limited information for estimating a specific factor after accounting for the general distress factor. The substantial overlap between the proposed dimensions may further contribute to difficulties in obtaining a stable solution. Thus, although a bifactor structure has been supported in previous research, the present findings do not provide sufficient evidence to evaluate this structure in our sample. Taken together, the findings support a unidimensional interpretation of the K6, consistent with previous studies supporting a one-factor structure [12, 16, 18].

The measurement invariance analyses provide further support for the use of the K6 across age groups and gender. Configural and metric invariance were supported across the age groups and between men and women, indicating that the one-factor structure and factor loadings were sufficiently comparable across these groups. These findings suggest that the K6 measures psychological distress in a comparable manner across age and gender, with the items showing comparable relationships with the underlying construct across groups. This is particularly relevant given the broad age range of the present population and the potential for differences in the way psychological distress is expressed or reported across demographic groups. Overall, the findings strengthen the evidence for the use of the K6 in population-based studies involving comparisons across age and gender. A key strength of this study is its very large and population-based sample (*n* ≈ 31,800) from LH, which was regularly conducted in five regions in Mid-Sweden using established procedures for data collection. The large, population-based sample from five regions in Mid-Sweden provided broad population coverage within the participating regions and enabled analyses across age groups and gender.

There are, however, also limitations to this study. First, the cross-sectional design limits the ability to draw conclusions about test–retest reliability, responsiveness to change, or predictive validity with respect to clinical outcomes. Second, as this study investigated reliability and validity according to classical test theory, we did not include analyses from item response theory, such as DIF. Third, self-reported survey responses also entail a risk of response bias, for example due to survey fatigue or non-response. Nevertheless, the LH adheres to established routines with reported response rates and dropout analyses, which increases transparency regarding representativeness. Fourth, mixed modes of data collection (i.e., postal and web) may introduce so-called mode effects, which were not possible to analyze in the data obtained for this study.

The findings support continued use of the K6 in Swedish population surveys such HLV and LH to monitor psychological distress over time and across groups. The K6 is, as shown, short, free of charge, and widely distributed, with documented ability to differentiate serious mental illness at the population level, thereby facilitating early identification and supporting resource planning in health care. In clinical contexts, the K6 can serve as a rapid screening tool. However, threshold values should be calibrated against Swedish reference data and, where possible, against clinical criteria (e.g., via ROC analyses) to optimize sensitivity and specificity, in line with international practice. In primary prevention follow-ups, potential floor effects must be considered, particularly in groups with low symptom burden, particularly among males as shown in this study, as shown in Fig. 1.

## Conclusions

The Swedish version of the K6 demonstrates strong reliability and functions in practice as a unidimensional measure of psychological distress in a large, population-based Swedish context. The scale enables comparable estimates across gender and age at the total-score level and is therefore well suited for monitoring psychological distress in public health surveys and as a rapid screening tool in clinical settings. Continued methodological development, particularly regarding DIF and calibration of threshold values against clinical diagnoses in Swedish context, is recommended to further strengthen the scale’s utility and precision in both population-based and clinical applications.

## Data Availability

The data that support the findings of this study are available from Liv och Hälsa. Restrictions apply to the availability of these data, which were used under license for this study.

## Abbreviations

CFA: Confirmatory factor analysis
CFI: Comparative fit index
DIF: Differential item functioning
HET: Health on Equal Terms
K6: The six item Kessler scale
K10: The ten item Kessler scale
LH: The Life and Health Survey
RMSEA: Root mean square error of approximation
SCB: Statistics Sweden
SD: Standard deviation
SRMR: Standardized root mean square residual
TLI: Tucker–Lewis index
WLSMV: Weighted least squares mean- and variance-adjusted estimator
